# Supplementary material for: Direct haplotype-resolved 5-base HiFi sequencing for genome-wide profiling of hypermethylation outliers in a rare disease cohort
Source: Nat Commun. 2023 May 29;14:3090. doi: 10.1038/s41467-023-38782-1 (PMC10226990; doi:10.1038/s41467-023-38782-1)
Supplement: Supplementary file 1 — Supplementary Information [file 41467_2023_38782_MOESM1_ESM.pdf]

Supplementary Figures and Tables

Direct haplotype-resolved 5-base HiFi sequencing for genome-wide profiling of hypermethylation outliers in a rare disease cohort

Warren A Cheung[1], Adam F Johnson[1], William J Rowell[2], Emily Farrow[1,3], Richard Hall[2], Ana SA Cohen[3,4], John C Means[1], Tricia N Zion[1], Daniel M Portik[2], Christopher T Saunders[2], Boryana Koseva[1], Chengpeng Bi[1], Tina K Truong[5], Carl Schwendinger-Schreck[1], Byunggil Yoo[1], Jeffrey J Johnston[1], Margaret Gibson[1], Gilad Evrony[5], William B Rizzo[6], Isabelle Thiffault[3,4], Scott T Younger[1,3], Tom Curran[7], Aaron M Wenger[2], Elin Grundberg[1,3]* and Tomi Pastinen[1,3]*

[1] Department of Pediatrics, Genomic Medicine Center, Children's Mercy Kansas City, Kansas City, MO
[2] Pacific Biosciences, Menlo Park, CA
[3] Department of Pediatrics, School of Medicine, University of Missouri Kansas City, Kansas City, MO
[4] Department of Pathology and Laboratory Medicine, Children's Mercy Kansas City, Kansas City, MO
[5] Center for Human Genetics and Genomics, Department of Pediatrics, Department of Neuroscience and Physiology, New York University Grossman School of Medicine, New York, USA
[6] Child Health Research Institute, Department of Pediatrics, Nebraska Medical Center, Omaha, NE
[7]Children's Mercy Research Institute, Kansas City, MO
*Correspondence to egrundberg@cmh.edu; tpastinen@cmh.edu

This PDF file includes:
Supplementary Figures 1-20
Supplementary Table 1

Table of Contents

Supplementary Figure 1………………………………………………………. Page 3
Supplementary Figure 2……………………………………………………….. Page 4
Supplementary Figure 3……………………………………………………….. Page 5
Supplementary Figure 4……………………………………………………….. Page 6
Supplementary Figure 5……………………………………………………….. Page 7
Supplementary Figure 6……………………………………………………….. Page 8
Supplementary Figure 7……………………………………………………….. Page 9
Supplementary Figure 8………………………………………………………..… Page 10
Supplementary Figure 9………………………………………………………..… Page 11
Supplementary Figure 10………………………………………………………… Page 12
Supplementary Figure 11………………………………………………………… Page 13
Supplementary Figure 12………………………………………………………… Page 14
Supplementary Figure 13………………………………………………………… Page 15
Supplementary Figure 14………………………………………………………… Page 16
Supplementary Figure 15………………………………………………………… Page 17
Supplementary Figure 16………………………………………………………… Page 18
Supplementary Figure 17………………………………………………………… Page 19
Supplementary Figure 18………………………………………………………… Page 20
Supplementary Figure 19………………………………………………………… Page 21
Supplementary Figure 20………………………………………………………… Page 22
Supplementary Table 1…………………………………………………………... Page 23

Supplementary Figure 1. Density plot of the distribution of CpG-based correlation. Correlation estimates (Pearson R, x-axis) of the top (N=500) variable CpGs obtained from 93 samples profiled by HiFi-GS and WGBS. Blue line represents CpG-based pair-wise measures of the 500 tested CpGs and red line represents similar number but when WGBS values are permuted.

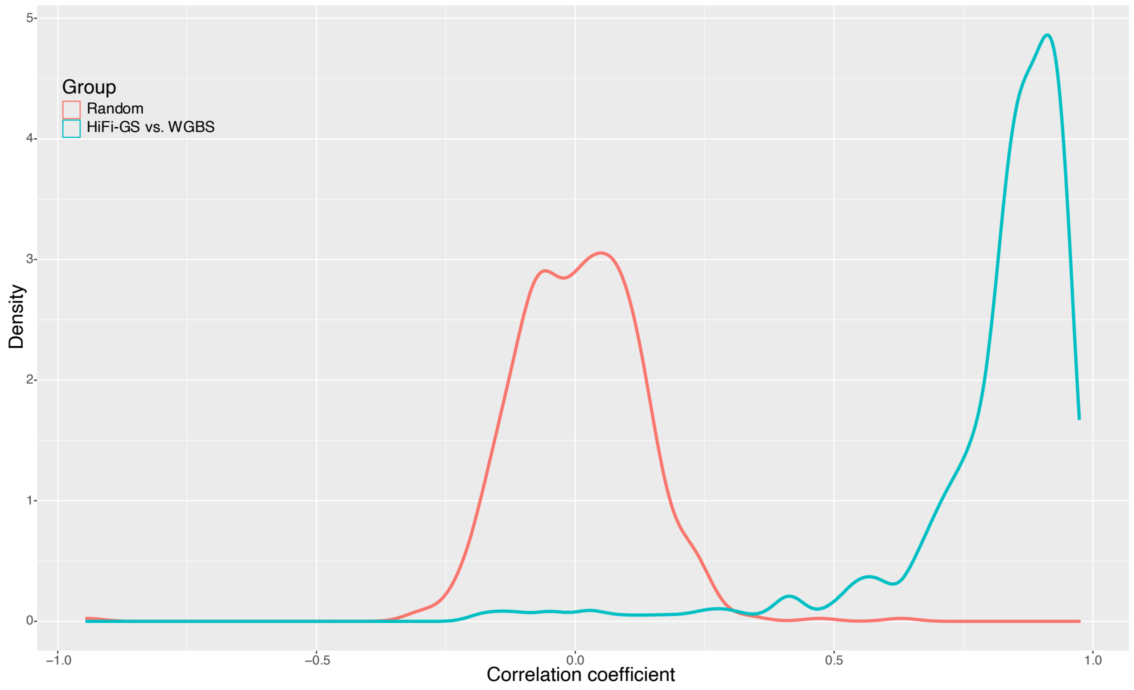

Supplementary Figure 2. Haplotype-resolved HiFi-GS of an imprinted region - *GNAS*. The *GNAS* locus (chr20: 58,851,237-58,851,799) is shown for a complete trio. Haplotype-resolved HiFi-GS reads are depicted with CpG modification coloring (blue indicating unmethylated CpG prediction and red indicating methylated CpG prediction with color intensity corresponding to base modification probabilities). Proband's Hap1 carries SNVs (black boxes) identifying it as paternally inherited matching paternal Hap2 which shows hypermethylation (red) pattern. However, proband's Hap1 shows hypomethylated (blue) pattern indicating maternal allele-specific hypermethylation in proband (Hap 2).

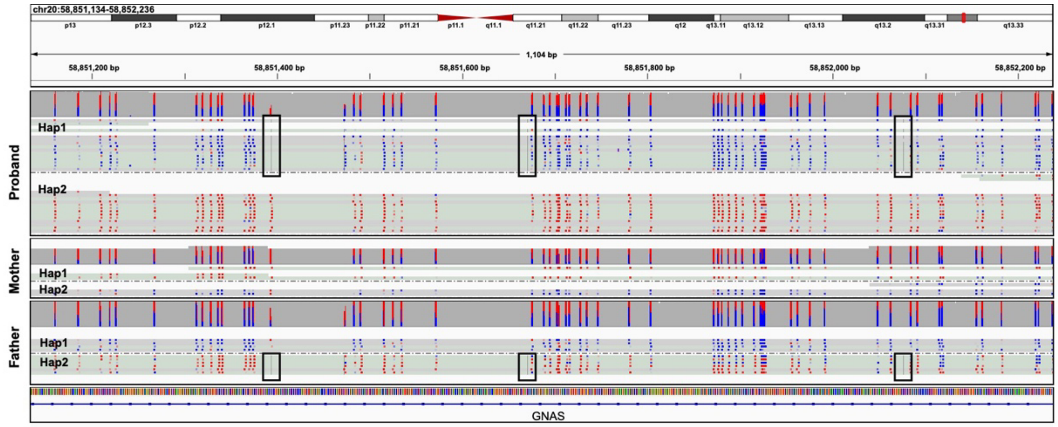

Supplementary Figure 3. Haplotype-resolved HiFi-GS of an imprinted region – *PLAGL1*. The *PLAGL1* locus (chr6:144,008,510-144,008,709) is shown for a complete trio. Haplotype-resolved HiFi-GS reads are depicted with CpG modification coloring (blue indicating unmethylated CpG prediction and red indicating methylated CpG prediction with color intensity corresponding to base modification probabilities). Proband's Hap2 carries SNVs (black boxes) identifying it as paternally inherited matching paternal Hap2 which shows hypermethylation (red) pattern. However, proband's Hap2 shows hypomethylated (blue) pattern indicating maternal allele-specific hypermethylation in proband (Hap1).

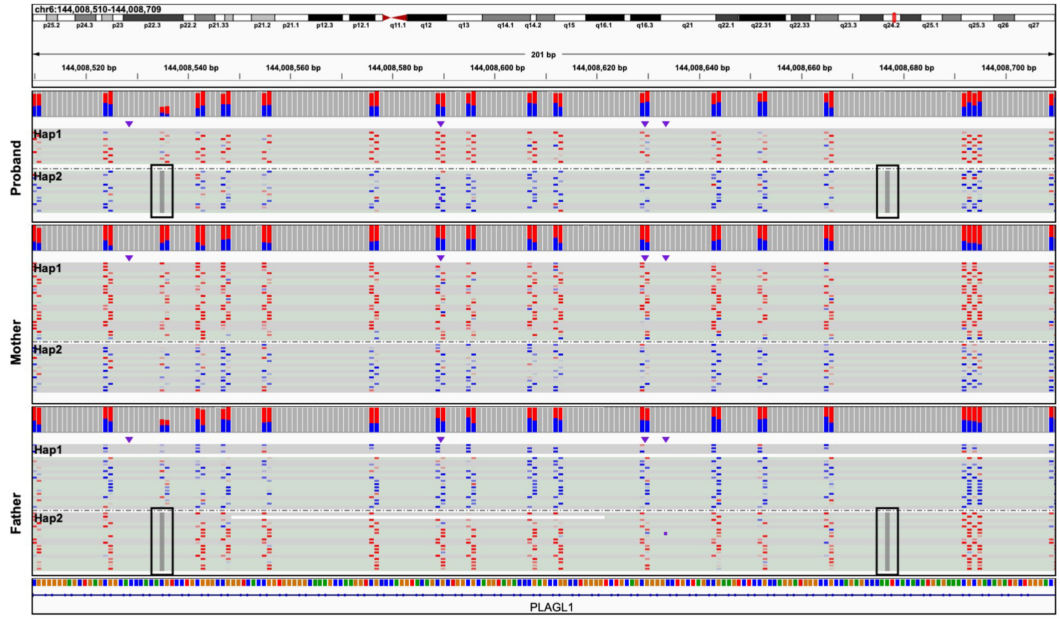

Supplementary Figure 4. Haplotype-resolved HiFi-GS of an imprinted region - *MAGEL2.* The *MAGEL2* locus (chr15: 23,648,164-23,648,386) is shown for a complete trio. Haplotype-resolved HiFi-GS reads are depicted with CpG modification coloring (blue indicating unmethylated CpG prediction and red indicating methylated CpG prediction with color intensity corresponding to base modification probabilities). Proband’s Hap2 carries SNVs (black boxes) identifying it as maternally inherited matching maternal Hap1 which shows hypomethylation (blue) pattern. However, proband’s Hap2 shows hypermethylated (red) pattern indicating paternal allele-specific hypermethylation in proband (Hap2).

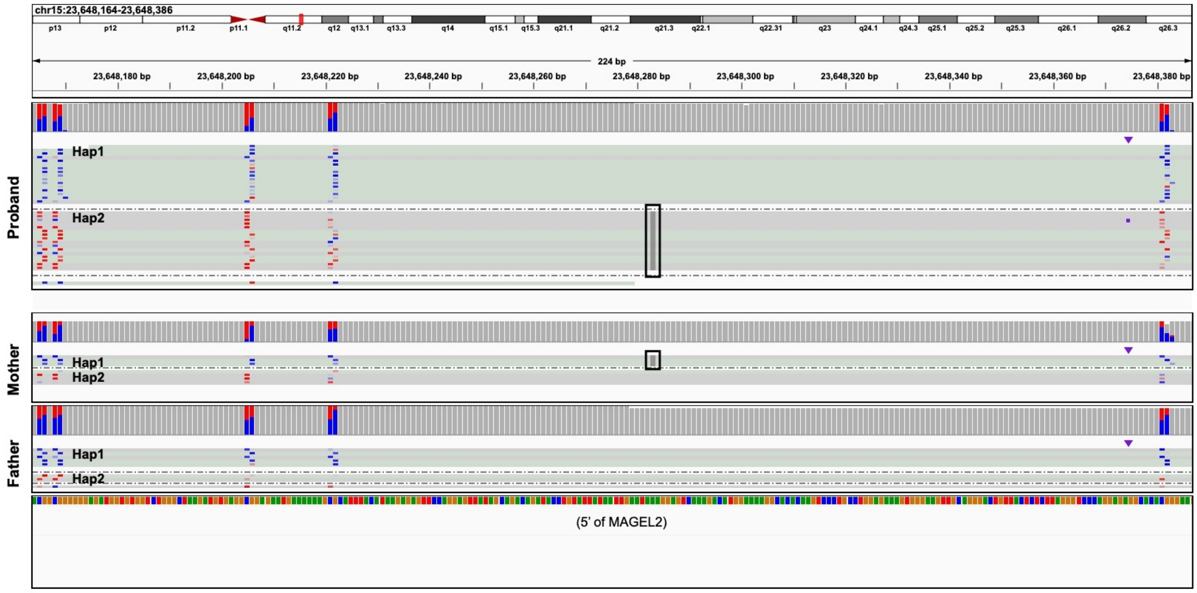

Supplementary Figure 5. Haplotype-resolved HiFi-GS of an imprinted region - *MEG3*. The *MEG3*/*DLM1* locus (chr14: 100,809,369-100,809,835) is shown for a complete trio. Haplotype-resolved HiFi-GS reads are depicted with CpG modification coloring (blue indicating unmethylated CpG prediction and red indicating methylated CpG prediction with color intensity corresponding to base modification probabilities). Proband's Hap1 carries SNVs (black boxes) identifying it as paternally inherited matching paternal Hap2 which shows hypomethylation (blue) pattern. However, proband's Hap1 shows hypermethylated (red) pattern indicating maternal allele-specific hypermethylation in proband (Hap1).

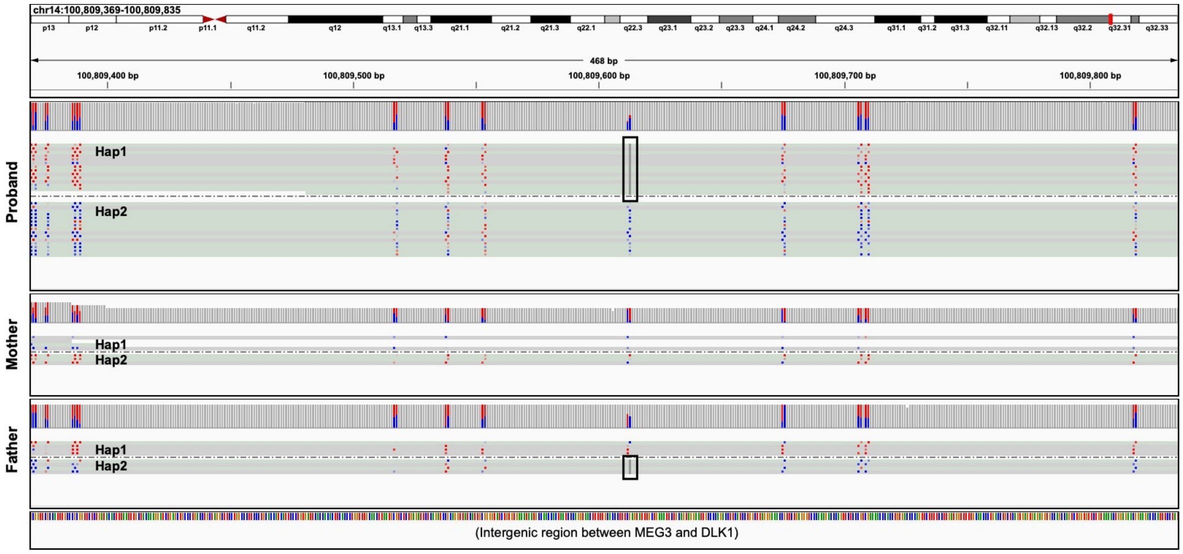

Supplementary Figure 6. Validation of HiFi-GS resolved allele-specific methylation by WGBS. Genomic view of an example of a rare SNV (black box) mapping in *cis* close to a hyper-mCpG tile on chromosome 10 associated with allele-specific hypermethylation. Track depicts haplotype-resolved HiFi-GS reads with CpG modification coloring (blue indicating unmethylated CpG prediction and red indicating methylated CpG prediction with color intensity corresponding to base modification probabilities) in proband carrying the rare A allele as well as an independent (control) sample homozygous for the common C allele. Hap 1 denotes haplotype 1 and Hap 2 denotes haplotype 2. Top tracks depict parallel assessment of CpG methylation (y-axis, 0-100%) by WGBS in the proband and an unrelated control sample not carrying the rare SNV.

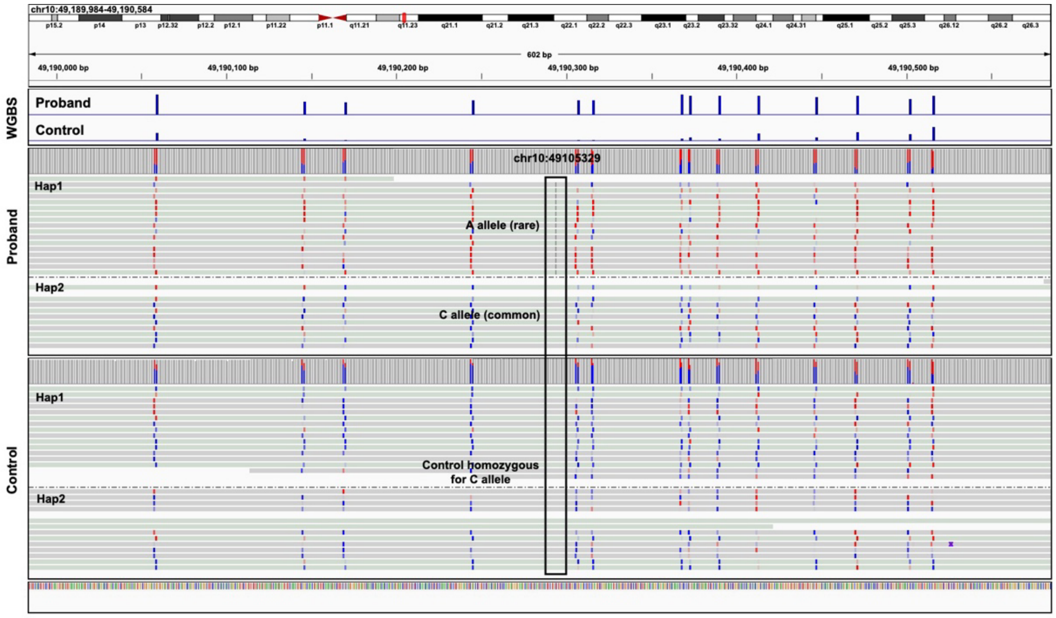

Supplementary Figure 7. Validation of HiFi-GS resolved allele-specific methylation by WGBS. Genomic view of an example of a rare SNV (black box) mapping in *cis* close to a hyper-mCpG tile on chromosome 22 associated with allele-specific hypermethylation. Track depicts haplotype-resolved HiFi-GS reads with CpG modification coloring (blue indicating unmethylated CpG prediction and red indicating methylated CpG prediction with color intensity corresponding to base modification probabilities). Hap 1 denotes haplotype 1 and Hap 2 denotes haplotype 2. Top tracks depict parallel assessment of CpG methylation (y-axis, 0-100%) by WGBS in the proband and an unrelated control sample not carrying the rare SNV.

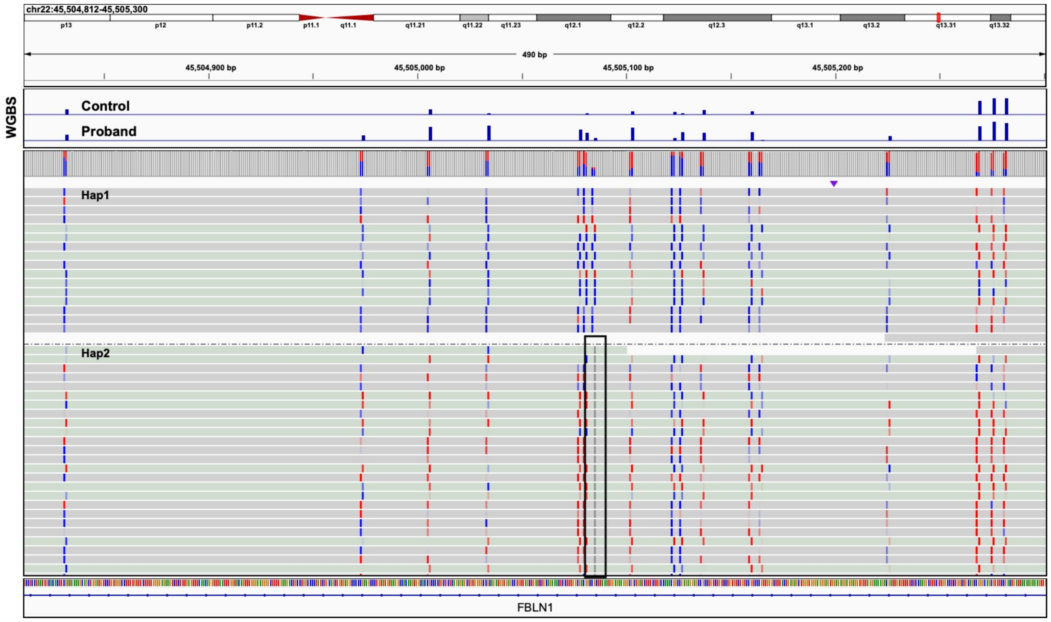

Supplementary Figure 8. Validation of HiFi-GS resolved allele-specific methylation by WGBS. Genomic view of an example of a rare SNV (black box) mapping in *cis* close to a hyper-mCpG tile on chromosome 10 associated with allele-specific hypermethylation. Track depicts haplotype-resolved HiFi-GS reads with CpG modification coloring (blue indicating unmethylated CpG prediction and red indicating methylated CpG prediction with color intensity corresponding to base modification probabilities). Hap 1 denotes haplotype 1 and Hap 2 denotes haplotype 2. Top tracks depict parallel assessment of CpG methylation (y-axis, 0-100%) by WGBS in the proband and an unrelated control sample not carrying the rare SNV.

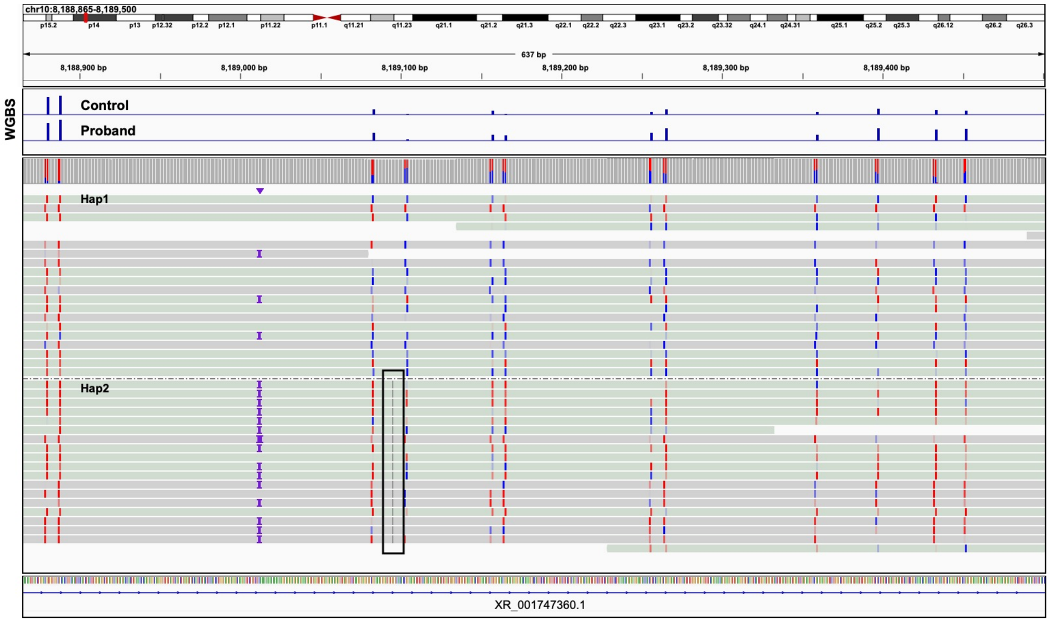

Supplementary Figure 9. **Short deletion and allele-specific methylation resolved by HiFi-GS.** Genomics view of 160 bp in an intergenic region comprising an 8 bp deletion (Proband 1 Hap1) resolved by HiFi-GS that results in proximal hypermethylation. Track depicts haplotype-resolved HiFi-GS reads with CpG modification coloring (blue indicating unmethylated CpG prediction and red indicating methylated CpG prediction with color intensity corresponding to base modification probabilities). Hap 1 denotes haplotype 1 and Hap 2 denotes haplotype 2. Top tracks depict parallel assessment of CpG methylation (y-axis, 0-100%) by WGBS in the proband and an unrelated control sample not carrying the deletion.

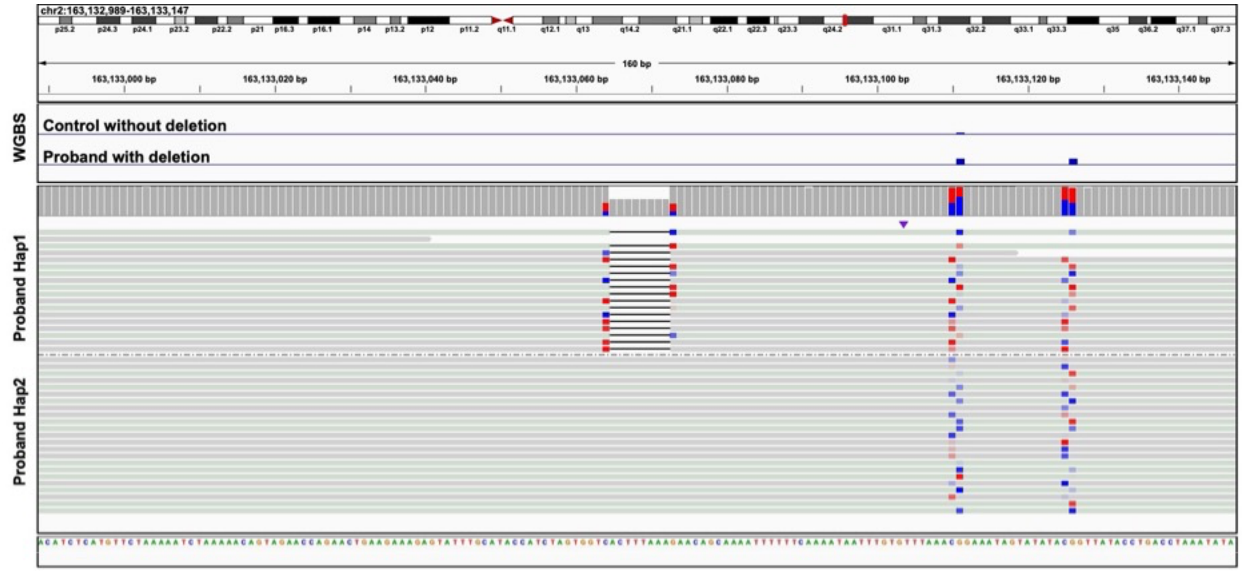

Supplementary Figure 10. Short deletion and allele-specific methylation resolved by HiFi-GS. For the autosomal recessive disease gene *NHLRC2*, an A to C transversion (chr10:113,854,859, black box) maps 900bp upstream of a rare hyper-mCpG tile (red box). To assess the size (in base pair) of the extreme hyper-CpG tile linked to the rare, local (*cis*) variant, HiFi-GS reads (bottom tracks) overlapping the candidate *cis*-variant were fetched. Track depicts haplotype-resolved HiFi-GS reads with CpG modification coloring (blue indicating unmethylated CpG prediction and red indicating methylated CpG prediction with color intensity corresponding to base modification probabilities). Top track depicts P-values (-log10, 0-11) from 2-by-2 Fisher's exact test examining each CpG state (methylated vs. unmethylated) in reads with rare C versus common A-allele carrying reads, respectively. Hypermethylation shows statistical significance for the phased C-allele from haplotype-resolved HiFi-GS data across ~200-300bp. Second and third track from the top depict average CpG methylation level (y-axis, 0-100%) across all C carrying reads (rare) measured by HiFi-GS.

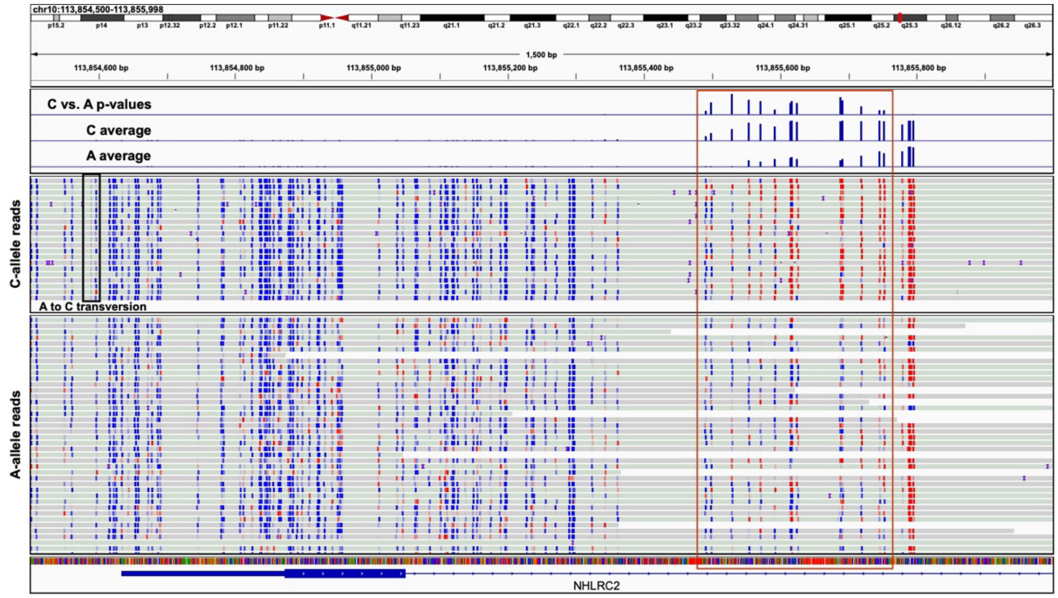

Supplementary Figure 11. Rare SNV and allele-specific methylation in regulatory element. A. Genomics view of 82 bp at an intergenic locus showing a rare SNV (blue box) that results in proximal hypermethylation in one allele (Hap2). Track depicts haplotype-resolved HiFi-GS reads with CpG modification coloring (blue indicating unmethylated CpG prediction and red indicating methylated CpG prediction with color intensity corresponding to base modification probabilities). Hap 1 denotes haplotype 1 and Hap 2 denotes haplotype 2. **B**. Zoomed out region in UCSC genome browser showing overlap of SNV (blue line) with *cis*-regulatory element (CRE) as mapped by ENCODE.

A

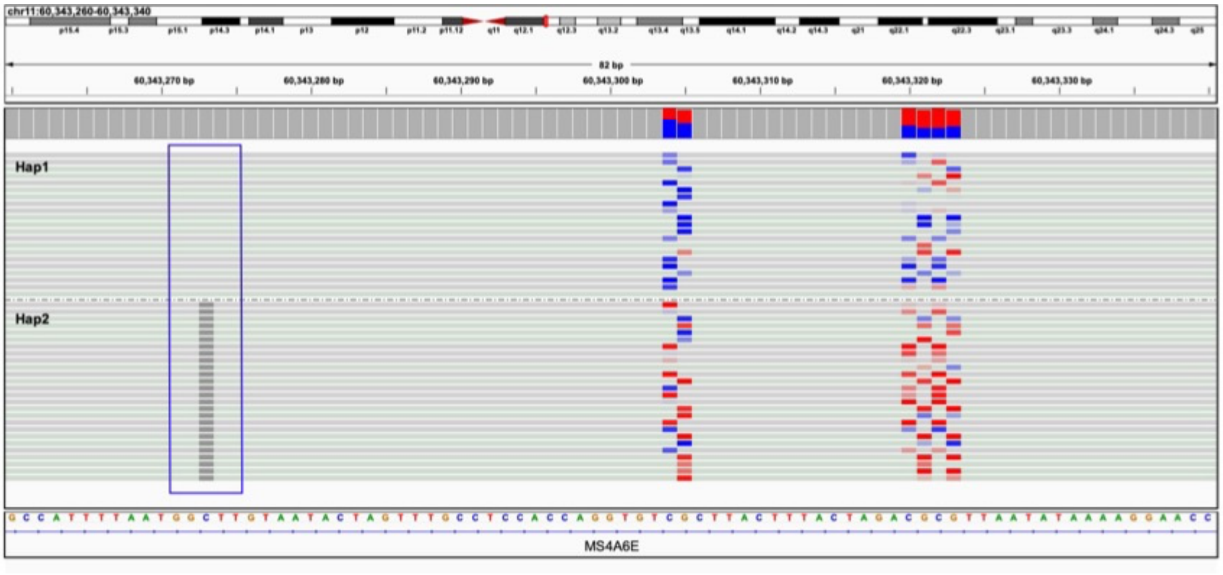

B

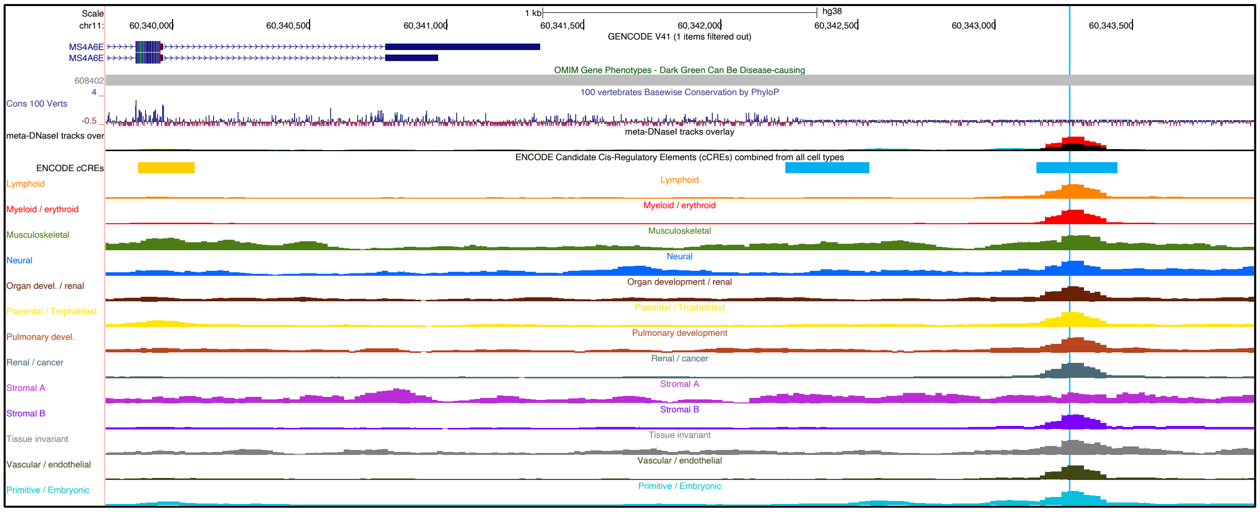

Supplementary Figure 12. Rare SV and allele-specific methylation in regulatory element. **A.** Genomics view of 13kb at the *LOC199882* locus comprising a breakpoint of a CNV (black box, Proband 2) that results in proximal hypermethylation. Lower tracks depict haplotype-resolved HiFi-GS reads with CpG modification coloring (blue indicating unmethylated CpG prediction and red indicating methylated CpG prediction with color intensity corresponding to base modification probabilities) in a proband with and without the CNV. Hap 1 denotes haplotype 1 and Hap 2 denotes haplotype 2. Upper tracks depict CpG methylation by WGBS (0-100%, y axis) in the same samples confirming hypermethylation in proband carrying the CNV. **B.** Zoomed out region in UCSC genome browser showing overlap of CNV (blue box) with *cis*-regulatory element (CRE) as mapped by ENCODE.

A

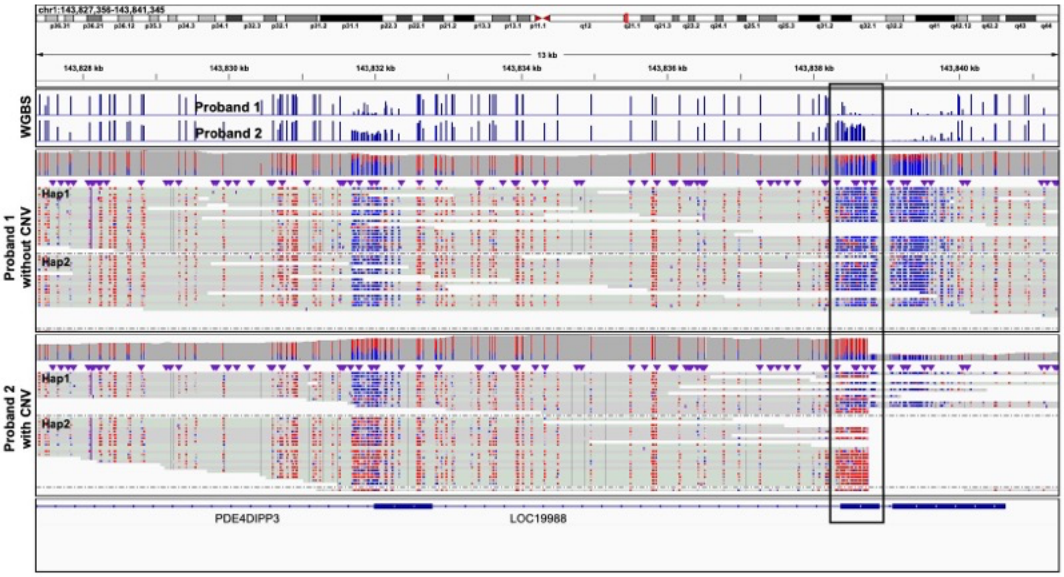

B

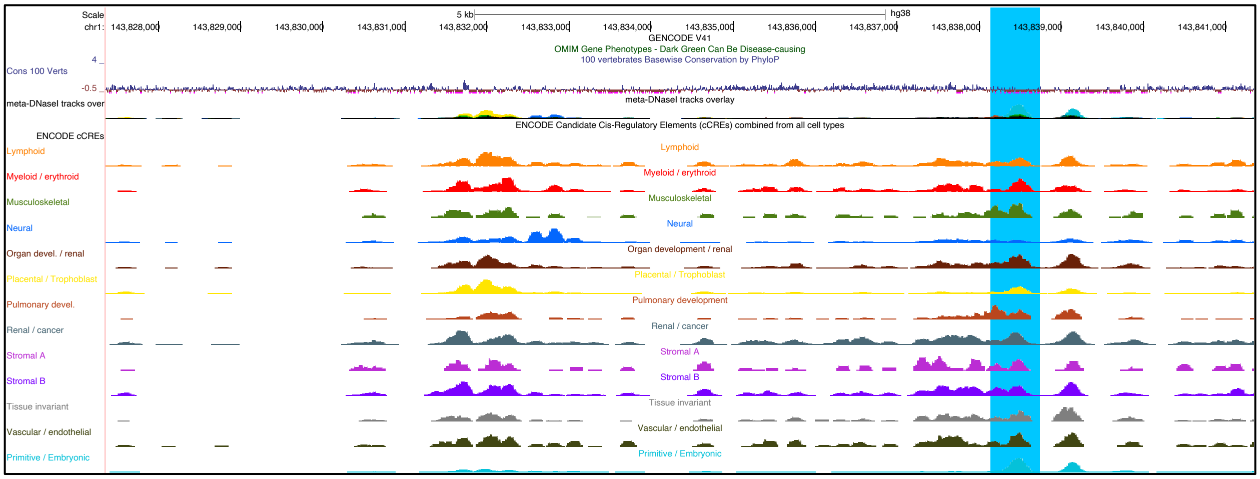

Supplementary Figure 13. Repeat expansion and allele-specific methylation in regulatory element. A. Identification of a previously uncharacterized repeat expansion at *ELF1* locus associated with hypermethylation. Tracks depict haplotype-resolved HiFi-GS reads with CpG modification coloring (blue indicating unmethylated CpG prediction and red indicating methylated CpG prediction with color intensity corresponding to base modification probabilities) in a proband with the repeat expansion. Hap 1 denotes haplotype 1 and Hap 2 denotes haplotype 2. **B.** Zoomed in region in UCSC genome browser showing overlap of repeat expansion (blue box) with *cis*-regulatory element (CRE) as mapped by ENCODE.

A

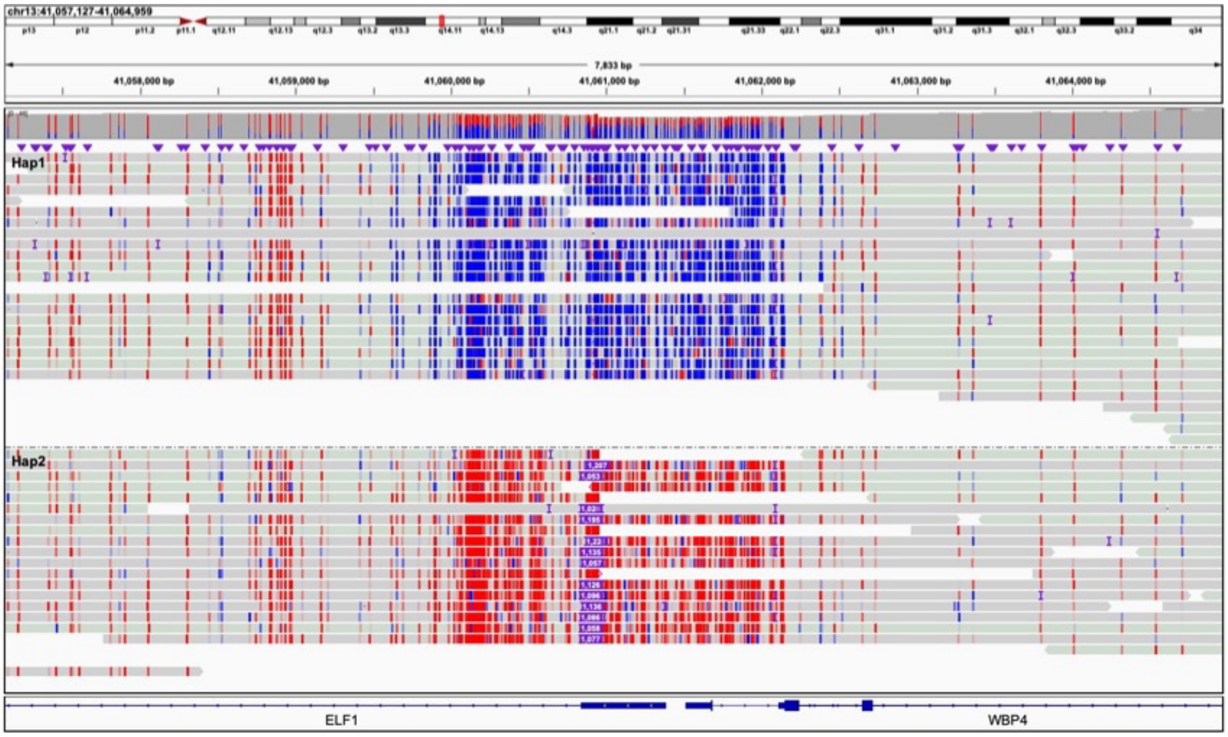

B

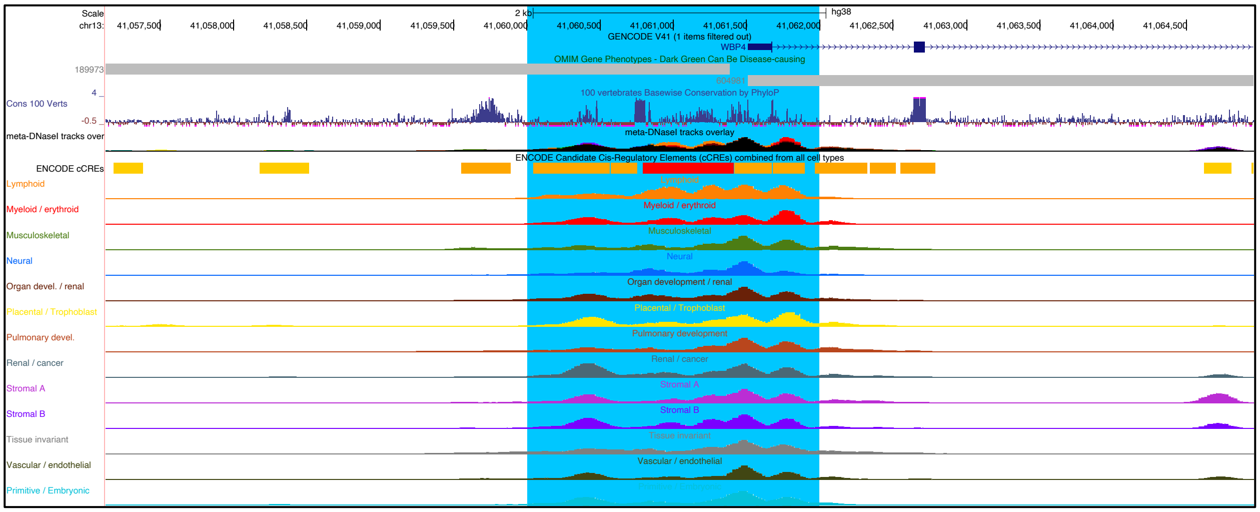

Supplementary Figure 14. Repeat expansion and allele-specific methylation in regulatory element. A. Identification of a previously uncharacterized repeat expansion at *LINGO3* locus associated with promoter hypermethylation. Tracks depict haplotype-resolved HiFi-GS reads with CpG modification coloring (blue indicating unmethylated CpG prediction and red indicating methylated CpG prediction with color intensity corresponding to base modification probabilities) in a proband with the repeat expansion. Hap 1 denotes haplotype 1 and Hap 2 denotes haplotype 2. **B.** Zoomed out region in UCSC genome browser showing overlap of repeat expansion (blue box) with *cis*-regulatory element (CRE) as mapped by ENCODE.

A.

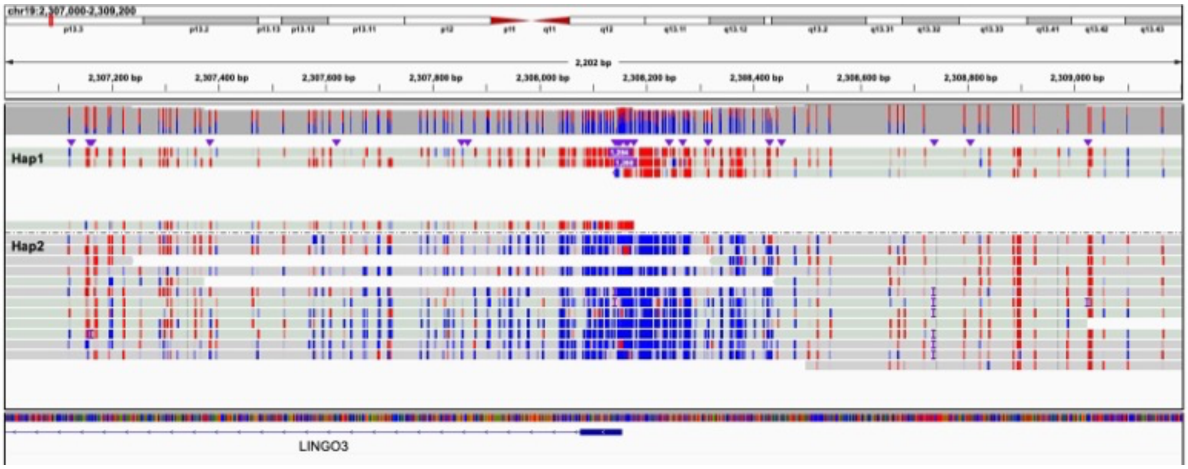

B.

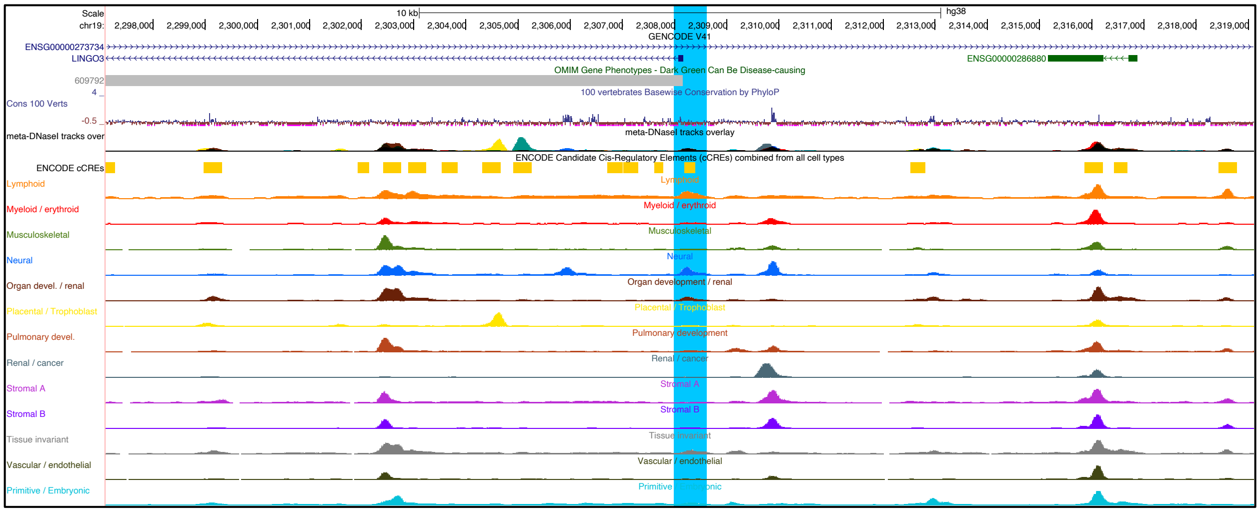

Supplementary Figure 15. Repeat expansion and allele-specific methylation in regulatory element. A. Identification of a previously uncharacterized repeat expansion at *SNED1* locus associated with hypermethylation. Tracks depict haplotype-resolved HiFi-GS reads with CpG modification coloring (blue indicating unmethylated CpG prediction and red indicating methylated CpG prediction with color intensity corresponding to base modification probabilities) in a proband with the repeat expansion. Hap 1 denotes haplotype 1 and Hap 2 denotes haplotype 2. **B.** Zoomed out region in UCSC genome browser showing overlap of repeat expansion (blue box) with *cis*-regulatory element (CRE) as mapped by ENCODE.

A

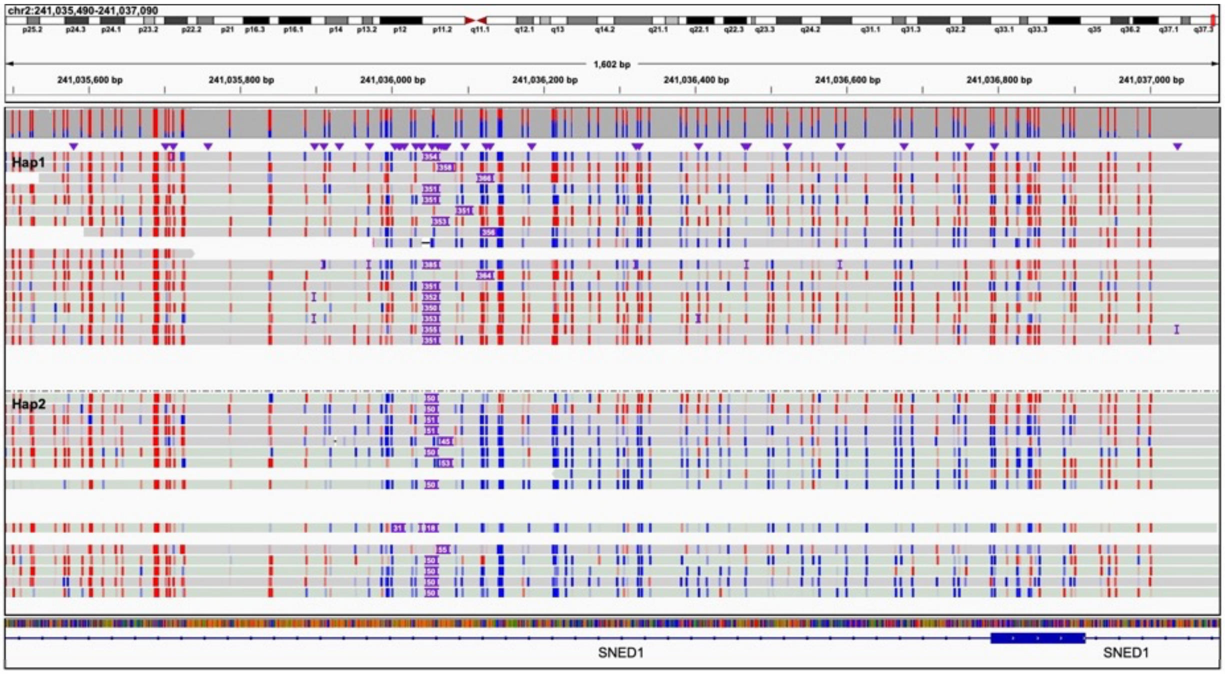

B

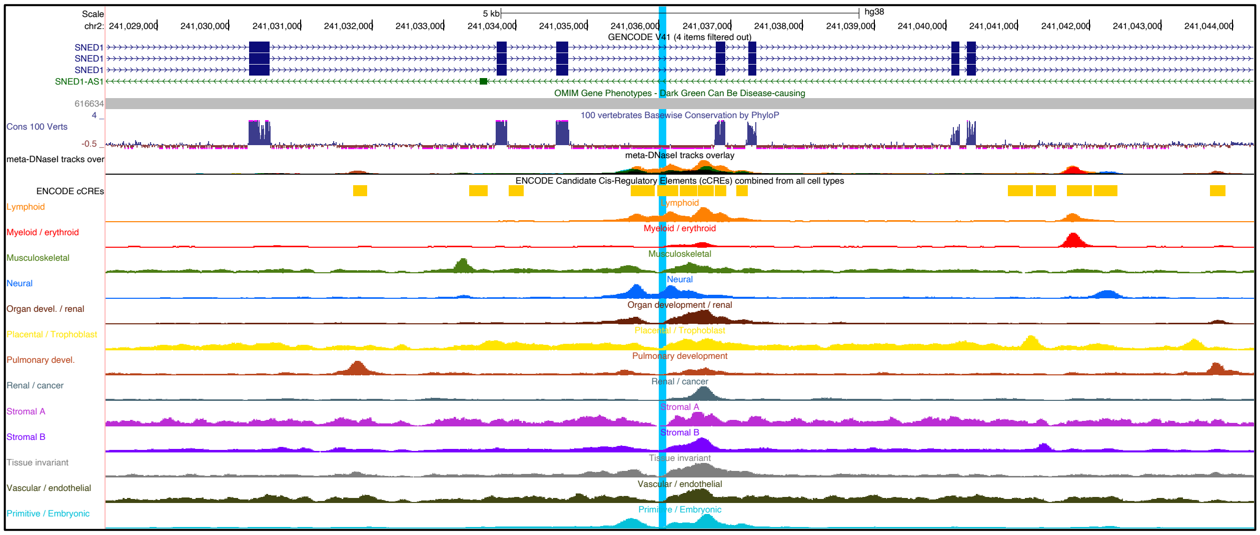

Supplementary Figure 16. Duplication and allele-specific methylation in regulatory element. A. Identification of a rare duplication (10.6kb, black box, solid line) associated with "compensatory" promoter hypermethylation (black box, dashed line) at the *COX20* disease locus. Lower tracks depict haplotype-resolved HiFi-GS reads with CpG modification coloring (blue indicating unmethylated CpG prediction and red indicating methylated CpG prediction with color intensity corresponding to base modification probabilities) in complete trio showing maternal inheritance of the duplication in proband. Hap 1 denotes haplotype 1 and Hap 2 denotes haplotype 2. Top tracks show parallel assessment of CpG methylation by WGBS (y-axis, 0-100%) validating hypermethylation associated with the duplication. **B.** Zoomed in region in UCSC genome browser showing overlap of hypermethylation effect (grey box) associated with the duplication (blue box) with *cis*-regulatory element (CRE) as mapped by ENCODE.

A

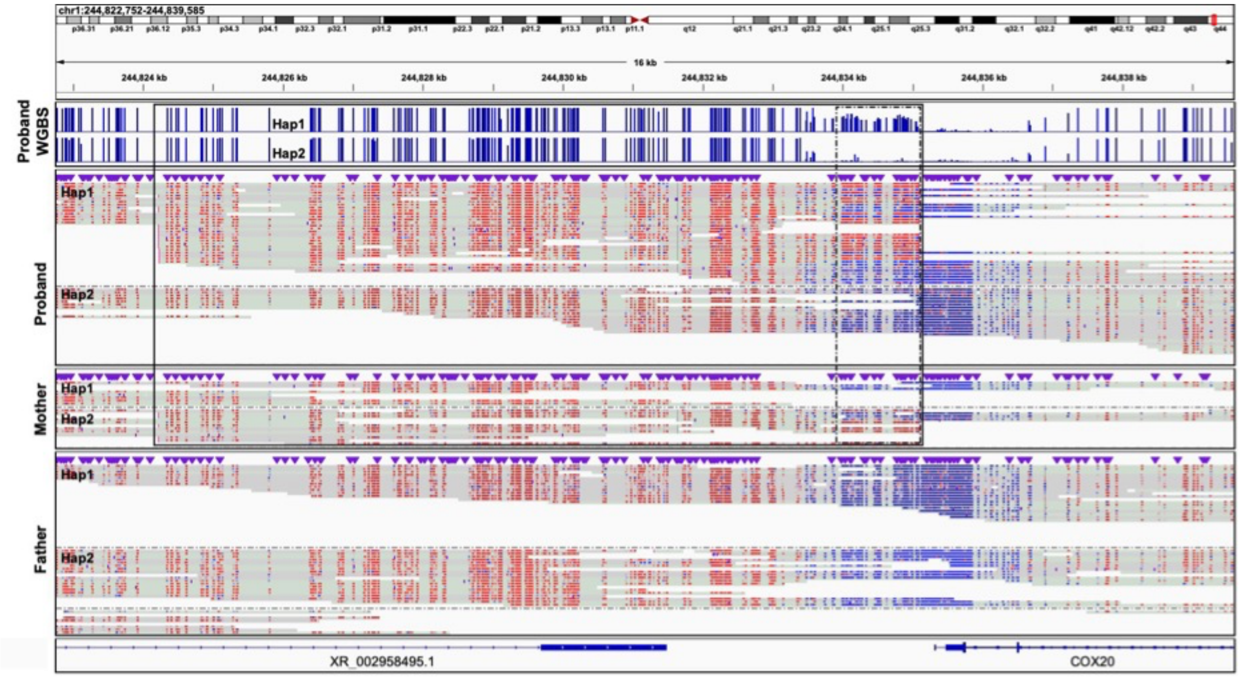

B

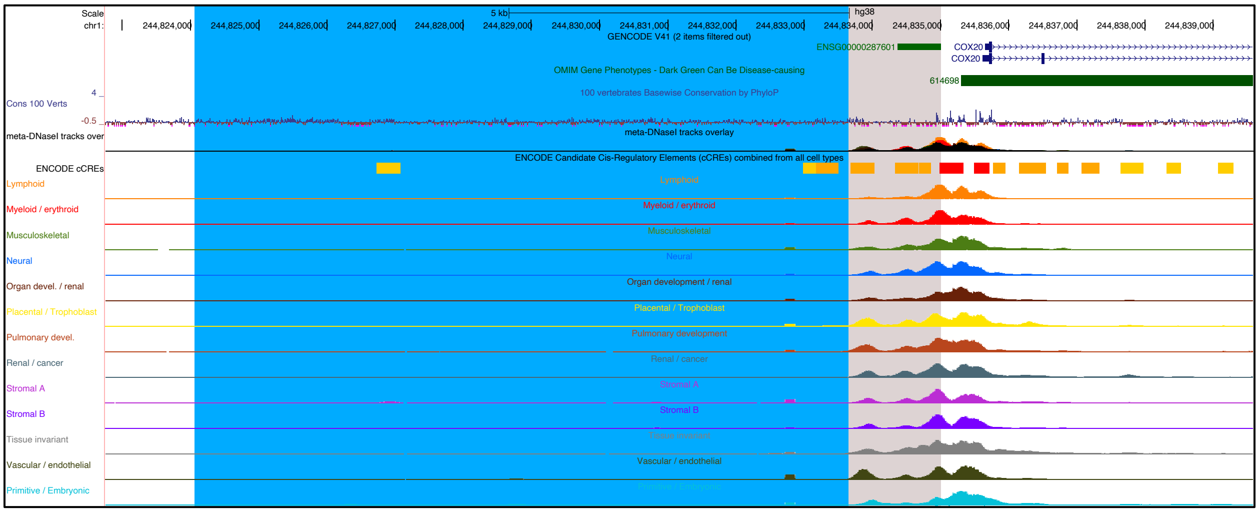

Supplementary Figure 17. Deletion and allele-specific methylation in regulatory element. **A.** Genomics view of ~3.5kb at the *RNF166* locus comprising an inherited deletion (Proband Hap2 and Mother Hap2) resolved by HiFi-GS that results in 2kb hypermethylation surrounding the deletion. Tracks depict HiFi-GS reads with CpG modification coloring (blue indicating unmethylated CpG prediction and red indicating methylated CpG prediction with color intensity corresponding to base modification probabilities) in proband and mother. Hap 1 denotes haplotype 1 and Hap 2 denotes haplotype 2. **B.** Zoomed in region in UCSC genome browser showing overlap of hypermethylation effect (light green box) associated with the deletion with *cis*-regulatory element (CRE) as mapped by ENCODE.

A

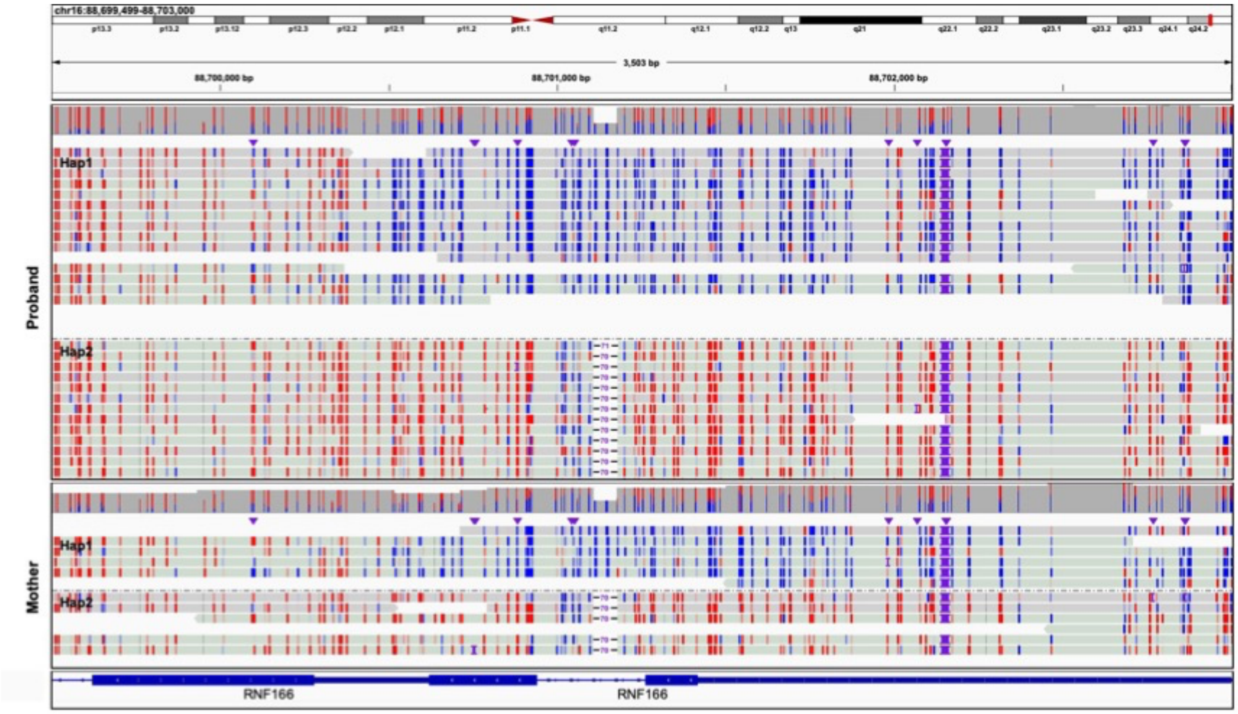

B

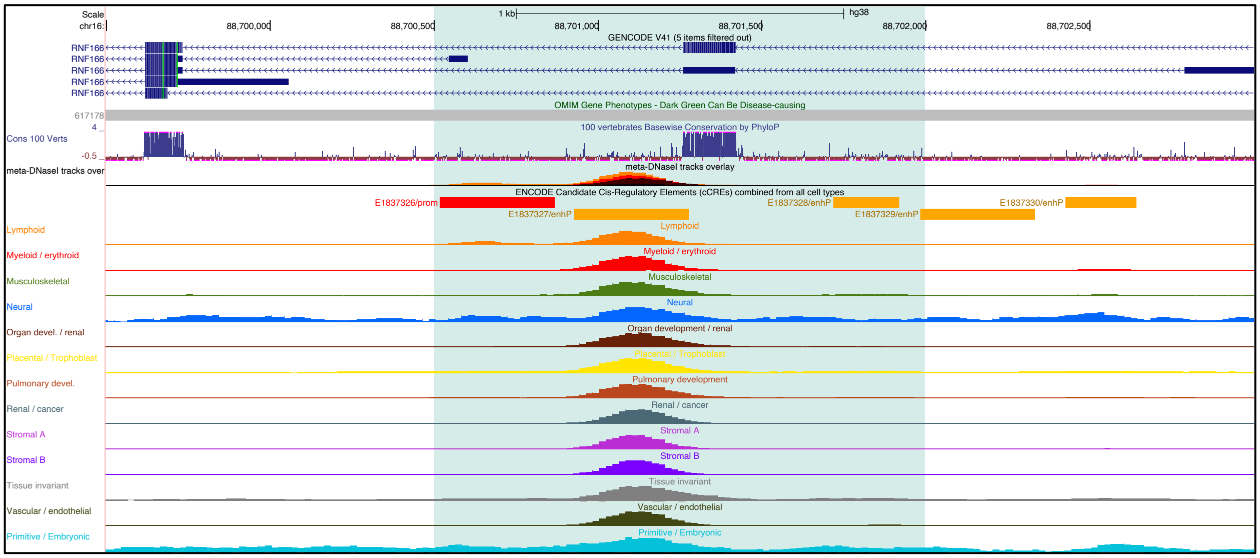

Supplementary Figure 18. Deletion and allele-specific methylation in regulatory element. **A.** Genomics view of ~300bp comprising a 13 bp deletion (blue box) shared in sibling (Sibling 1; Hap1 and Sibling 2; Hap2) that results in proximal hypermethylation. Lower tracks depict haplotype-resolved HiFi-GS reads with CpG modification coloring (blue indicating unmethylated CpG prediction and red indicating methylated CpG prediction with color intensity corresponding to base modification probabilities) in siblings and unrelated proband without the deletion. Hap 1 denotes haplotype 1 and Hap 2 denotes haplotype 2. Top tracks show parallel assessment of CpG methylation by WGBS (y-axis, 0-100%) validating hypermethylation associated with the deletion. **B.** Zoomed out region in UCSC genome browser showing overlap of hypermethylation effect (light green box) with *cis*-regulatory element (CRE) as mapped by ENCODE.

A

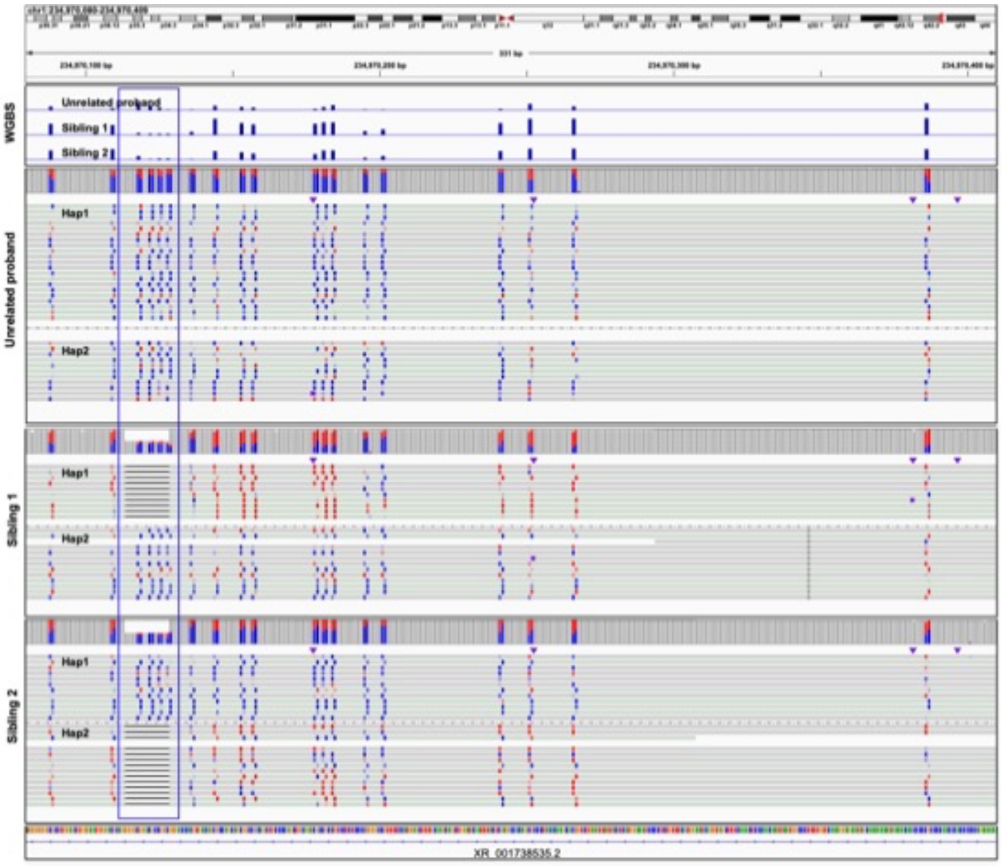

B

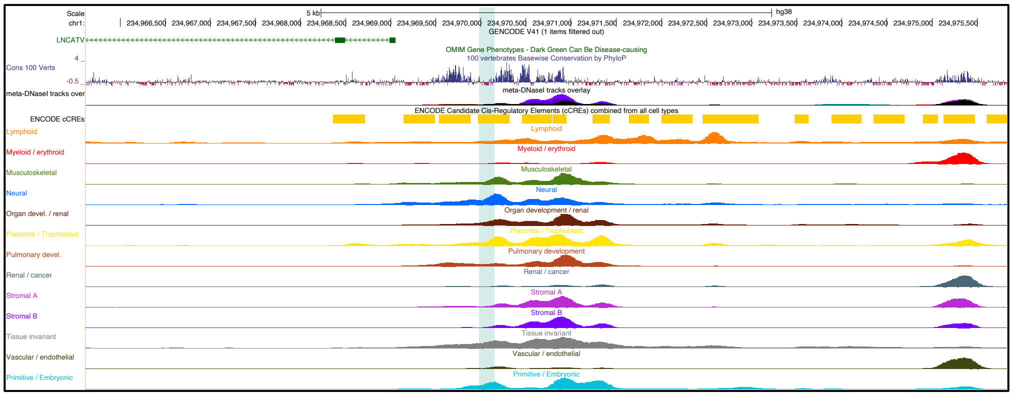

Supplementary Figure 19. Validation of allele-specific methylation by full-length cDNA sequencing A. Genomics view of the *FES* locus showing differential expression of T and G alleles (red box; two-sided Chi-square test, P=0.008). **B.** Genomics view of the *NUP153* locus showing differential expression of A and G alleles (red box; two-sided Chi-square test, P=0.005). Tracks depict full length cDNA (IsoSeq) sequence reads generated from proband-specific blood-derived iPSC lines.

A

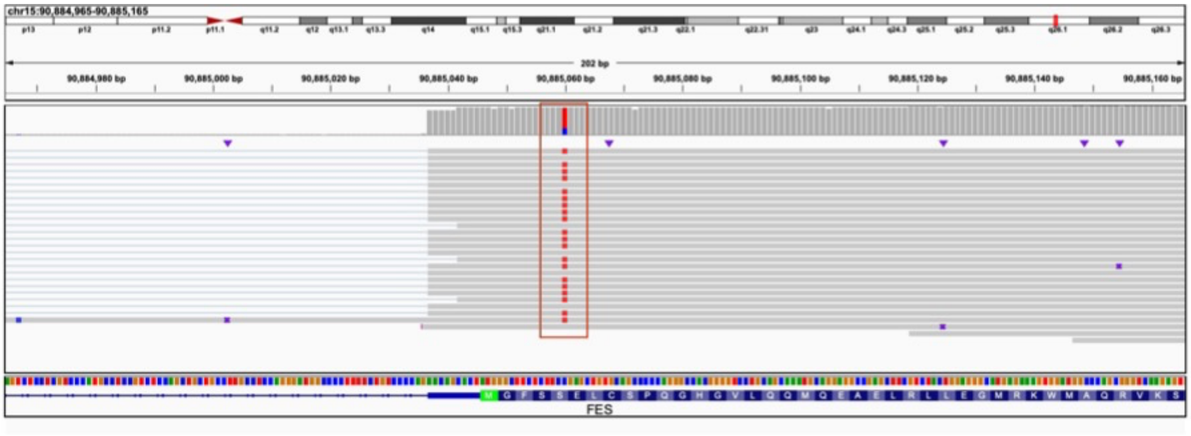

B

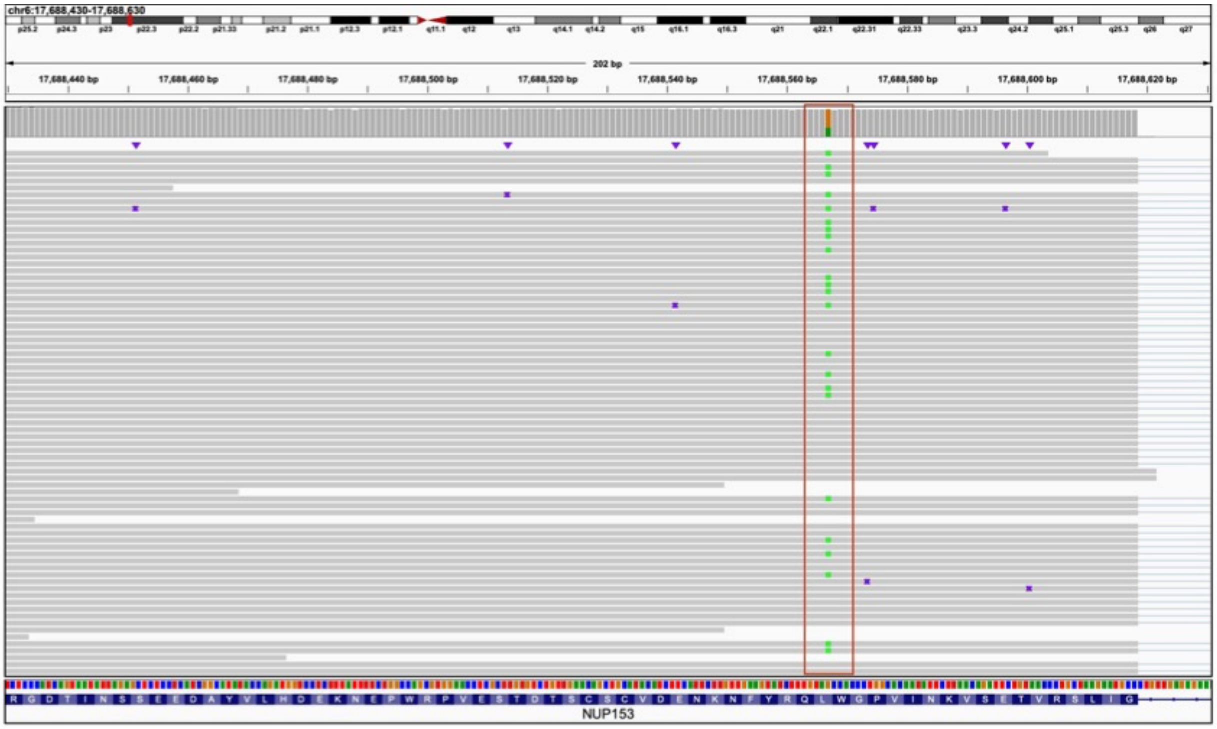

Supplementary Figure 20. Bulk gene expression across tissues for *GNAO1*. GTEx Analysis Release V8 (dbGaP Accession phs000424.v8.p2) was used to study gene expression of *GNAO1* (ENSG00000087258.14) across multiple tissue types. Expression values (y axis) are shown in TPM (transcripts per million) calculated from a gene model with isoforms collapsed to a single gene. Violin plots are shown as median and 25th and 75th percentiles; points are displayed as outliers if they are above or below 1.5 times the interquartile range. The following samples sizes were used: Adipose – Subcutaneous, N=663; Adipose - Visceral (Omentum), N=341; Adrenal Gland, N=258; Artery – Aorta, N=432; Artery – Coronary, N=240; Artery – Tibial, N=663; Bladder, N=21; Brain – Amygdala, N=152; Brain - Anterior cingulate cortex (BA24), N=176; Brain - Caudate (basal ganglia), N=246; Brain - Cerebellar Hemisphere, N=215; Brain – Cerebellum, N=241; Brain – Cortex, N=255; Brain - Frontal Cortex (BA9), N=209; Brain – Hippocampus, N=197; Brain – Hypothalamus, N=202; Brain - Nucleus accumbens (basal ganglia), N=246; Brain - Putamen (basal ganglia), N=205; Brain - Spinal cord (cervical c-1), N=159; Brain - Substantia nigra, N=139; Breast - Mammary Tissue, N=459; Cells - Cultured fibroblasts, N=504; Cells - EBV-transformed lymphocytes, N=174; Cervix – Ectocervix, N=9; Cervix – Endocervix, N=10; Colon – Sigmoid, N=373; Colon – Transverse, N=406; Esophagus - Gastroesophageal Junction, N=375; Esophagus – Mucosa, N=555; Esophagus – Muscularis, N=515; Fallopian Tube, N=9; Heart - Atrial Appendage, N=429; Heart - Left Ventricle, N=432; Kidney – Cortex, N=85; Kidney – Medulla, N=4; Liver, N=226; Lung, N=578; Minor Salivary Gland, N=162; Muscle – Skeletal, N=803; Nerve – Tibial, N=619; Ovary, N=180; Pancreas, N=328; Pituitary, N=283; Prostate, N=245; Skin - Not Sun Exposed (Suprapubic), N=604 Skin - Sun Exposed (Lower leg), N=701; Small Intestine - Terminal Ileum, N=187; Spleen, N=241; Stomach, N=359; Testis, N=361; Thyroid, N=653; Uterus, N=142; Vagina, N=156; Whole Blood, N=755

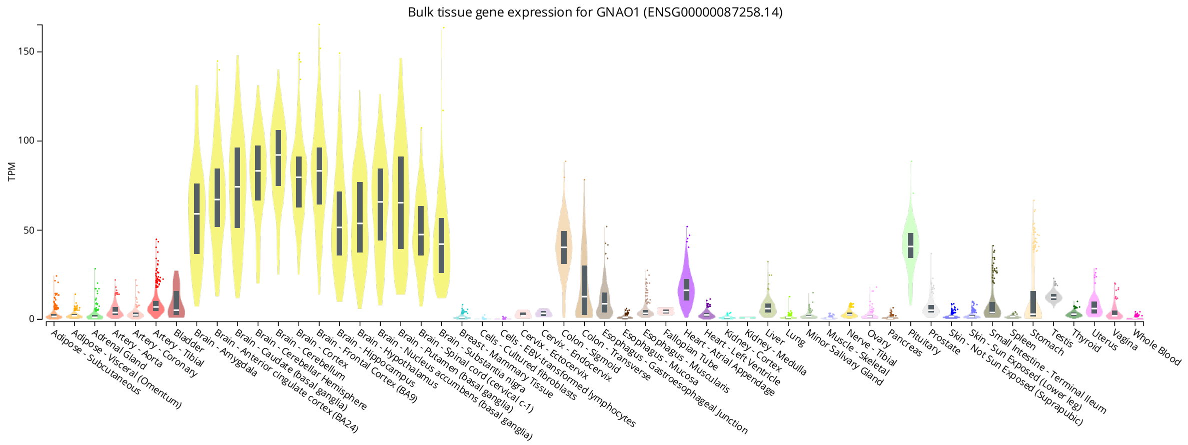

Supplementary Table 1: Differential expression of transcripts near hyper-mCpG outliers

Chr	Gene	Pos	Allele 1	Allele 2	Allele1 total read count:case	Allele2 total read count:case	Allele freq. (case)	Allele1 total read count:ctrl	Allele2 total read count:ctrl	Allele freq. (ctrl)	Chi^2 P value
6	NUP153	17688567	G	A	44	21	0.68	137	151	0.48	0.005
15	FES	90885060	C	T	14	49	0.22	32	38	0.46	0.008
13	SKA3	21161859	T	C	48	28	0.63	214	223	0.49	0.031
6	CENPW	126340356	G	T	60	50	0.55	20	35	0.36	0.042
10	ARHGAP21	24584440	C	G	17	32	0.35	181	177	0.51	0.053
5	JAKMIP2	147644946	C	T	123	106	0.54	480	553	0.46	0.056
1	LRIF1	110951572	C	T	81	48	0.63	509	419	0.55	0.108
10	ECHS1	133373302	A	G	88	80	0.52	150	99	0.60	0.136
15	TP53BP1	43475576	G	C	87	112	0.44	1367	1484	0.48	0.279
1	PDPN	13583922	C	T	21	33	0.39	254	284	0.47	0.305
17	VPS53	562535	G	A	9	5	0.64	52	60	0.46	0.329
3	EMC3	9986610	G	A	26	37	0.41	45	46	0.49	0.403
12	ESPL1	53268840	C	A	213	155	0.58	130	110	0.54	0.413
19	GRWD1	48446029	G	T	20	16	0.56	12	15	0.44	0.536
2	PLCL1	198085516	G	A	5	8	0.38	62	58	0.52	0.540
7	IFRD1	112472224	G	A	35	25	0.58	781	675	0.54	0.560
3	HPS3	149145364	A	G	12	10	0.55	154	176	0.47	0.620
10	TRDMT1	17162188	G	A	24	32	0.43	409	467	0.47	0.675
6	TAPBP	33305078	G	C	40	41	0.49	224	260	0.46	0.691
1	DNM3	171987663	C	T	12	8	0.60	28	24	0.54	0.837
20	CD93	23084572	G	A	8	7	0.53	10	12	0.45	0.892
2	HNMT	138002079	C	T	104	89	0.54	15	13	0.54	1.000
