# Supplementary material for: Direct haplotype-resolved 5-base HiFi sequencing for genome-wide profiling of hypermethylation outliers in a rare disease cohort
Source: Nat Commun. 2023 May 29;14:3090. doi: 10.1038/s41467-023-38782-1 (PMC10226990; doi:10.1038/s41467-023-38782-1)
Supplement: Supplementary file 3 — Description of Additional Supplementary Files [file 41467_2023_38782_MOESM3_ESM.pdf]

Description of Additional Supplementary Files

File Name: Supplementary Data 1
Description: Overview of data set

File Name: Supplementary Data 2
Description: List of coverage of HiFi-GS samples included in the study

File Name: Supplementary Data 3
Description: Sample-wise correlation data of methylation levels across methods

File Name: Supplementary Data 4
Description: Characteristics of extreme hyper-mCpG tiles seen in 2 or less unrelated individuals

File Name: Supplementary Data 5
Description: Number of extreme hyper-mCpG tiles per individual

File Name: Supplementary Data 6
Description: Characteristics of extreme hyper-mCpG tiles with large z-score

File Name: Supplementary Data 7
Description: Number of extreme hyper-mCpG tiles with large z score per individual

File Name: Supplementary Data 8
Description: Characteristics of extreme hyper-mCpG tiles in affected patients only
