# Supplementary material for: Direct haplotype-resolved 5-base HiFi sequencing for genome-wide profiling of hypermethylation outliers in a rare disease cohort
Source: Nat Commun. 2023 May 29;14:3090. doi: 10.1038/s41467-023-38782-1 (PMC10226990; doi:10.1038/s41467-023-38782-1)
Supplement: Supplementary file 5 — Reporting Summary [file 41467_2023_38782_MOESM5_ESM.pdf]

nature portfolio

Corresponding author(s): Elin Grundberg and Tomi Pastinen

Last updated by author(s): May 5, 2023

Reporting Summary

Nature Portfolio wishes to improve the reproducibility of the work that we publish. This form provides structure for consistency and transparency in reporting. For further information on Nature Portfolio policies, see our Editorial Policies and the Editorial Policy Checklist.

Statistics

For all statistical analyses, confirm that the following items are present in the figure legend, table legend, main text, or Methods section.

n/a	Confirmed	
☐	☒	The exact sample size (*n*) for each experimental group/condition, given as a discrete number and unit of measurement
☐	☒	A statement on whether measurements were taken from distinct samples or whether the same sample was measured repeatedly
☐	☒	The statistical test(s) used AND whether they are one- or two-sided<br>*Only common tests should be described solely by name; describe more complex techniques in the Methods section.*
☐	☒	A description of all covariates tested
☐	☒	A description of any assumptions or corrections, such as tests of normality and adjustment for multiple comparisons
☐	☒	A full description of the statistical parameters including central tendency (e.g. means) or other basic estimates (e.g. regression coefficient) AND variation (e.g. standard deviation) or associated estimates of uncertainty (e.g. confidence intervals)
☐	☒	For null hypothesis testing, the test statistic (e.g. *F*, *t*, *r*) with confidence intervals, effect sizes, degrees of freedom and *P* value noted<br>*Give P values as exact values whenever suitable.*
☒	☐	For Bayesian analysis, information on the choice of priors and Markov chain Monte Carlo settings
☒	☐	For hierarchical and complex designs, identification of the appropriate level for tests and full reporting of outcomes
☐	☒	Estimates of effect sizes (e.g. Cohen's *d*, Pearson's *r*), indicating how they were calculated

Our web collection on statistics for biologists contains articles on many of the points above.

Software and code

Policy information about availability of computer code

Data collection

No software used for data collection

Data analysis

No custom codes or software were used.

Circular consensus reads were generated with ccs v6.3 (https://github.com/PacificBiosciences/ccs) using the "--hifi-kinetics" option to generate consensus kinetics tags, and primrose v1.1 (https://github.com/PacificBiosciences/primrose) was used to predict 5mC modification of each CpG motif and generate base modification ("MM") and base modification probability ("ML") BAM tags. HiFi Read mapping, variant calling, and genome assembly were performed using a Snakemake workflow (https://github.com/PacificBiosciences/pb-human-wgs-workflow-snakemake). HiFi reads were mapped to GRCh38 (GCA_000001405.15) with pbmm2 v1.9 (https://github.com/PacificBiosciences/pbmm2). Structural variants were called with pbsv v2.8 (https://github.com/PacificBiosciences/pbsv) with "--hifi --tandem-repeats human_GRCh38_no_alt_analysis_set.trf.bed" options to pbsv discover and "--hifi -m 20" options to pbsv call. Small variants were called with DeepVariant v1.3 following DeepVariant best practices for PacBio reads (https://github.com/google/deepvariant/blob/r1.3/docs/deepvariant-pacbio-model-case-study.md) and locally phased with WhatsHap v1.0 32. Local phase haplotype tags ("HP") were added to the aligned BAM by WhatsHap v1.0. Pileup-based consensus methylation sites and probabilities were generated by the script "aligned_bam_to_cpg_scores.py" from pb-CpG-tools v1.1.0 (https://github.com/PacificBiosciences/pb-CpG-tools/) with the "-q 1 -m denovo -p model -c 10" options. Reads were visualized in IGV version 2.15.2.

Whole genome bisulfite sequence reads were pre-processed with fastp to trim adapter and low-quality bases, then alignment was performed with Illumina's DRAGEN aligner followed by post-processing with samtools 1.9, Picard 2.17.8, Bismark_v0.20.0 and Bis-SNP 0.82.2 for marking of duplicates, methylation calling and SNV calling. To avoid potential biases in downstream analyses, we applied our benchmark filtering criteria as follows; ≥ 5 total reads, no overlap with SNPs (dbSNP 137), ≤ 20% methylation difference between strands, no overlap with DAC

Blacklisted Regions (DBRs) or Duke Excluded Regions (DERs) generated by the ENCODE project: (http://hgwdev.cse.ucsc.edu/cgi-bin/hgFileUi?db=hg19&g=wgEncodeMapability). Methylation values at each site were calculated as total (forward and reverse) non-converted C-reads over total (forward and reverse) reads. CpGs were counted once per location combining both strands together.

For manuscripts utilizing custom algorithms or software that are central to the research but not yet described in published literature, software must be made available to editors and reviewers. We strongly encourage code deposition in a community repository (e.g. GitHub). See the Nature Portfolio guidelines for submitting code & software for further information.

Data

Policy information about availability of data

All manuscripts must include a data availability statement. This statement should provide the following information, where applicable:

- Accession codes, unique identifiers, or web links for publicly available datasets
- A description of any restrictions on data availability
- For clinical datasets or third party data, please ensure that the statement adheres to our policy

The 5-base HiFi-GS and WGBS raw and processed data generated in this study have been deposited in the dbGAP (https://www.ncbi.nlm.nih.gov/gap/) database under accession code phs002206.v4.p1 [https://www.ncbi.nlm.nih.gov/projects/gap/cgi-bin/study.cgi?study_id=phs002206.v4.p1]. Raw and processed data are available under restricted access due to IRB regulations and informed consent limiting access to users studying genetic diseases. Data access are provided by dbGAP (https://dbgap.ncbi.nlm.nih.gov/aa/wga.cgi?page=login) for certified investigators with local IRB approval in place. The reference genome GRCh38 (GCA_000001405.15) used in this study is available at ftp://ftp.ncbi.nlm.nih.gov/genomes/all/GCA/000/001/405/GCA_000001405.15_GRCh38/seqs_for_alignment_pipelines.ucsc_ids/GCA_00001405.15_GRCh38_no_alt_analysis_set.fna.gz. WGS and HTG sequences that are not part of the human reference genome, GRCh38 (including the ALT sequences) was added using https://www.ncbi.nlm.nih.gov/assembly/GCA_000786075.2/. The Gnomad v2.1 used in this study is available via Nirvana 3.18.1 (https://github.com/Illumina/Nirvana/).

Human research participants

Policy information about studies involving human research participants and Sex and Gender in Research.

Reporting on sex and gender	Sex is reported for all study participants and assigned from self-reporting at enrollment and confirmed with genomic analysis. We focus all analysis on autosomes thus the findings are applicable to both sexes and no sex-specific analysis was performed.
Population characteristics	The study cohort described includes 1243 affected probands from 1078 families, with a total of 1367 individuals (detailed in Supplemental Data 1) enrolled in the Genomic Answers for Kids program. Probands age at enrollment ranged from 0 to 32 years (median 6 years) with 47% being female and 53% male, respectively.
Recruitment	A patient is considered eligible for the study if clinical genetic testing is indicated or was previously completed, or if they have a suspected genetic diagnosis based on clinical presentation and/or an existing molecular or cytogenetic finding. Providers introduce the study and ask if the family is interested in the study. If the family is interested a study team member contacts the patient to arrange for informed consent. If possible, the family will be consented during their clinic appointment. If consent during the clinic appointment is not possible, the patient is contacted and enrolled over the phone at a time that is convenient. Study coordinators access the medical record and extract clinical information which will be kept indefinitely. Families may also self-refer using a QR code provided on study marketing materials, or by e-mailing the study group directly. Self-referred patients are screened for eligibility by a genetic counselor (GS) or similarly qualified study member to ensure no self-selection bias exist. If a self-referred patient does not qualify for the study based on clinical assessment by the GC or qualified study member, the family will be notified and encouraged to recontact the study if the patient's clinical presentation changes in the future. Self-referred patients who qualify for the study will be contacted and enrolled in the same way as those patients referred by a provider. If the family decides to no longer be part of the study, their information will be de-identified, but their de-identified information will remain.
Ethics oversight	The study complies with all relevant ethical regulations as approved by the Children's Mercy Institutional Review Board (IRB) (Study # 11120514). Informed written consent was obtained from all participants prior to study inclusion and included consenting for collecting biospecimens for the purpose of deriving patient-specific cell lines. Participants were not compensated for study participation.

Note that full information on the approval of the study protocol must also be provided in the manuscript.

Field-specific reporting

Please select the one below that is the best fit for your research. If you are not sure, read the appropriate sections before making your selection.

☒ Life sciences ☐ Behavioural & social sciences ☐ Ecological, evolutionary & environmental sciences

For a reference copy of the document with all sections, see nature.com/documents/nr-reporting-summary-flat.pdf

Life sciences study design

All studies must disclose on these points even when the disclosure is negative.

Sample size	No statistical method were used to predetermine the sample size. The sample size (173 patients from 152 families) was deemed sufficient for identification of methylation outliers rare enough to be considered candidate for Mendelian disease (allele frequency below 0.3%).
Data exclusions	No sample or data sets were excluded
Replication	To verify the reproducibility of the findings the following was performed:<br>1) orthogonal validation of long-read methylation profiling by whole-genome bisulfite sequencing in matched samples. This validation step was performed in 93 independent samples. All attempts showed high correlation across methods with the minimum, maximum and median correlation corresponding to r=0.77, r=0.92 and r=0.90, respectively. Individual (disaggregated) results are presented in Supplementary Data 3.<br>2) clinical validation for pathogenic variants. This validation step was performed using two different types of PCR reactions and conditions as outlined in the Methods section and a negative control sample was included showing no evidence of repeat expansion. The size of the repeat expansion in affected probands obtained from PCR validation was correlated with estimated size by HiFi Genome sequencing showing high correlation in all cases.<br>3) isoform profiling in matched samples for validation of cellular phenotype. This validation step was performed in 8 unrelated patients and in 68% of the cases a differential abundance of the allelic copies of the tested transcript beyond a 45:55 ratio was identified. Individual (disaggregated) results are presented in Supplementary Table 1.
Randomization	No experimental groups were included, thus no randomizations were done
Blinding	Family-based (trio) analysis is key for the identification of disease variants in suspected rare disease, thus no blinded analysis was performed.

Reporting for specific materials, systems and methods

We require information from authors about some types of materials, experimental systems and methods used in many studies. Here, indicate whether each material, system or method listed is relevant to your study. If you are not sure if a list item applies to your research, read the appropriate section before selecting a response.

Materials & experimental systems

n/a	Involved in the study
☒	☐ Antibodies
☒	☐ Eukaryotic cell lines
☒	☐ Palaeontology and archaeology
☒	☐ Animals and other organisms
☒	☐ Clinical data
☒	☐ Dual use research of concern

Methods

n/a	Involved in the study
☒	☐ ChIP-seq
☒	☐ Flow cytometry
☒	☐ MRI-based neuroimaging

March 2021
